# Application of Surgical Decision Model for Patients With Childhood Cataract: A Study Based on Real World Data

**DOI:** 10.3389/fbioe.2021.657866

**Published:** 2021-08-26

**Authors:** Jingjing Chen, Yifan Xiang, Longhui Li, Andi Xu, Weiling Hu, Zhuoling Lin, Fabao Xu, Duoru Lin, Weirong Chen, Haotian Lin

**Affiliations:** ^1^State Key Laboratory of Ophthalmology, Zhongshan Ophthalmic Center, Sun Yat-sen University, Guangzhou, China; ^2^Center of Precision Medicine, Sun Yat-sen University, Guangzhou, China

**Keywords:** childhood cataract, real-world data, surgical type, model validation, rare disease

## Abstract

Reliable validated methods are necessary to verify the performance of diagnosis and therapy-assisted models in clinical practice. However, some validated results have research bias and may not reflect the results of real-world application. In addition, the conduct of clinical trials has executive risks for the indeterminate effectiveness of models and it is challenging to finish validated clinical trials of rare diseases. Real world data (RWD) can probably solve this problem. In our study, we collected RWD from 251 patients with a rare disease, childhood cataract (CC) and conducted a retrospective study to validate the CC surgical decision model. The consistency of the real surgical type and recommended surgical type was 94.16%. In the cataract extraction (CE) group, the model recommended the same surgical type for 84.48% of eyes, but the model advised conducting cataract extraction and primary intraocular lens implantation (CE + IOL) surgery in 15.52% of eyes, which was different from the real-world choices. In the CE + IOL group, the model recommended the same surgical type for 100% of eyes. The real-recommended matched rates were 94.22% in the eyes of bilateral patients and 90.38% in the eyes of unilateral patients. Our study is the first to apply RWD to complete a retrospective study evaluating a clinical model, and the results indicate the availability and feasibility of applying RWD in model validation and serve guidance for intelligent model evaluation for rare diseases.

## Introduction

The application of artificial intelligence (AI) in medicine has achieved significant progress in medical researches (Crea, 2020; Xiang et al., 2020; Mervis, 2021). In most reported studies, medical AI systems perform excellently in both internal and external validations (Lin et al., 2018; Gurovich et al., 2019; Topol, 2019; Yamashita et al., 2021). However, the performances of AI systems in real-world applications are below expectations with much lower accuracies than the reported results (Lin H. et al., 2019; Baylor et al., 2020; Lee et al., 2021). More valid and exact methods are necessary to verify the effectiveness of the real application of medical AI systems to translate AI into clinical practice more safely (Cabitza et al., 2020; Lin and Yu, 2020).

A real-world clinical study (RWCS) was carried out to assess AI performance in clinical practice (Johnston et al., 2019; Nagendran et al., 2020), which is more objective and close to real application. However, the conduct of RWCS has executive risks for the indeterminate effectiveness of AI systems, especially in diagnosis (Sinha et al., 2020; Zhang et al., 2020) and therapy assistance (Schurink et al., 2005; Skrede et al., 2020). In addition, the RWCS probably takes a long time to validate clinical models for rare diseases, as it is not possible to accumulate enough cases in a short term for intelligent system evaluation.

Real-world data (RWD) can probably solve the executive risks of RWCSs. RWD is from patient medical chart reviews and registries rather than conventional randomized controlled trials (Elliott et al., 2016; Goldstein et al., 2019), which has been acknowledged as more favorable and valuable for guiding medical decisions (Goulooze et al., 2020). Published study has achieved a surgical decision model for a rare disease, childhood cataract (CC) (Lin D. et al., 2019). In our study, we collected RWD from 251 patients with CC and conducted a retrospective study to validate the CC surgical decision model. Our study applied RWD to complete a “retrospective RWCS” for model evaluation for the first time, and the results indicate the availability and feasibility of the application of RWD in model validation and serve as a guidance for AI system evaluation for rare diseases.

## Materials and Methods

A retrospective study was conducted from December 2018 to June 2020 at the Zhongshan Ophthalmic Center (ZOC), Guangdong, China. The RWD was collected from a national project for CC treatment and research, the Childhood Cataract Program of the Chinese Ministry of Health (CCPMOH) (Lin D. et al., 2019). This study followed the tenets of the Declaration of Helsinki and was approved by the Institutional Review Board of the ZOC at Sun Yat-sen University (IRB-ZOC-SYSU).

### RWD Collection

Only patients diagnosed with CC and registered in CCPMOH were enrolled. The inclusion criteria were as follows: patients registered at CCPMOH (1) who were under the age of 18 years; (2) who were diagnosed with CC in the first year after birth; (3) who had surgical treatment at ZOC; (4) who had complete medical data before and after surgical treatment; and (5) for whom written informed consent was obtained from the legal guardian. The exclusion criteria were as follows: patients (1) who were diagnosed with CC complicated with other ocular lesions; and (2) who were diagnosed with other ophthalmic diseases.

The RWD included sex, laterality, axial length, anterior photography, surgical age, surgical type, surgical laterality and other examination and therapy information. The surgical plans were all designed and performed by three cataract professors (Yizhi Liu, WC, and HL). The primary surgical types included cataract extraction (CE) and cataract extraction combined with intraocular lens implantation (CE + IOL). Posterior continuous curvilinear capsulorhexis and anterior vitrectomy surgical procedures were also performed in CC patients younger than 6 years old at the time of surgery. All CC patients were regularly followed up at 1 day, 1 week, 1 month, 3 months, and 6 months postoperatively. At each follow-up, the best corrected visual acuity (BCVA), ocular pressure (non-contact tonometer, TX-F, Canon, Tokyo, Japan), and anterior photography were collected. The children unable to cooperate with ocular pressure examination and anterior photography were sedated with 10% chloral hydrate (0.6–0.8 ml per kilogram, oral or clyster) and tested with a Tonopen contact electronic tonometer (Reichert Inc., United States) and a slit-lamp (BX900, HAAG-STRETT, Switzerland) to record the occurrence of postoperative complications.

### CC Surgical Type Decision Model

The CC surgical type decision model was established based on the data of 2421 CC patients recruited over 10 years from 1 January 2005 to 31 December 2014 (Lin D. et al., 2019). The original research aimed to provide timings of CE and IOL implantation for CC patients based on large-scale clinical experience, and to serve as a guidance for ophthalmologists to make treatment strategies. The model aimed to help choose the surgical type between CE and CE + IOL for CC patients.

Logit(P)=7.929-0.096×age+0.612×laterality-0.317×axial⁢length

Notes: Age in months; laterality: 1 for bilateral patients and 0 for unilateral patients; axial length in mm. Logit (*P*) ≥ 0.5 suggested CE; Logit (*P*) < 0.5 suggested CE + IOL. The model behaved well in the internal validation and obtained an AUC of 0.96 [95% confidence interval (CI): 0.94–0.97] and a Youden index of 0.86.

### Model Evaluation

We input the collected RWD into the surgical model to assess the consistency of the real surgical type and model-recommended surgical type. In addition, we compared the endpoint BCVAs, and the rates of complications between the real-recommended matched patients and the unmatched patients.

## Results

A total of 398 eyes of 251 patients with CC were enrolled. In total, 139 patients were male, and 112 patients were female. The mean follow-up time was 10.87 months [standard deviation (SD): 6.26 months]. The characteristics of RWD are shown in Table 1. There were 100 patients undergoing CE and 151 patients undergoing CE + IOL. The mean ages at surgery were 10.71 and 59.52 months in the two groups, respectively. The average endpoint BCVAs at 3 months after surgery were 0.52 ± 0.29 and 0.30 ± 0.28 for the right eyes of bilateral patients and the diseased eyes of unilateral patients, respectively.

**TABLE 1:** The characteristics of real-world data of enrolled patients with CC.

Characteristics	CE group	CE + IOL
Number	100	151
Male/Female	47/53	92/59
Age (month)	10.71 ± 13.455	59.52 ± 32.325
Bilateral/Unilateral	74/26	73/78
Eyes	174	224
Axial length (mm)	19.04 ± 1.97	22.39 ± 2.04
Endpoint BCVA (snellen)	0.10 ± 0.07	0.45 ± 0.30
Ocular hypertension	6/174	6/224
PCO	5/174	1/224

*CE, cataract extraction; CE + IOL, cataract extraction combined with intraocular lens implantation; CC, childhood cataract; BCVA, best corrected visual acuity; PCO, posterior capsule opacification.*

**FIGURE 1:**
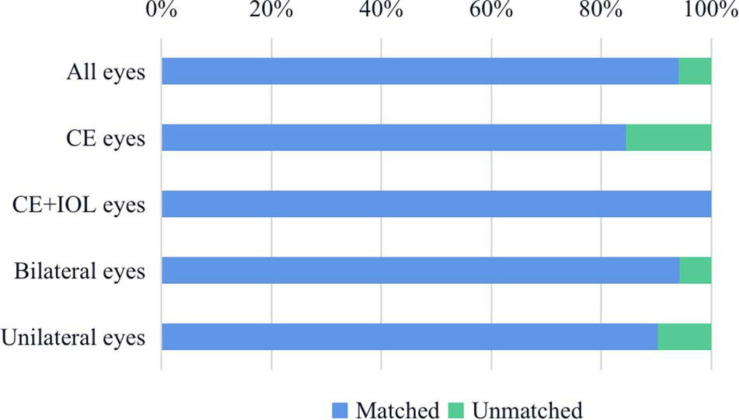
The consistency of the real surgical type and recommended surgical type. In the CE group, the model recommended the same surgical type for 84.48% of eyes; In the CE + IOL group, the model recommended the same surgical type for 100% of eyes; In the eyes of bilateral patients, the model recommended the same surgical type for 94.22% of eyes; In the eyes of unilateral patients, the model recommended the same surgical type for 90.38% of eyes. CE, cataract extraction; CE + IOL, cataract extraction combined with intraocular lens implantation.

The consistency of the real surgical type and model-recommended surgical type was 94.16% (Figure 1). In the CE group, the model recommended the same surgical type for 84.48% of eyes. In 15.52% of eyes, the model advised conducting CE + IOL surgery, which was different from the real-world choices. In the CE + IOL group, the model recommended the same surgical type for 100% of eyes. The real-recommended matched rates were 94.22% in the eyes of bilateral patients and 90.38% in the eyes of unilateral patients.

There were 27 eyes of 20 patients (17 eyes of 10 bilateral patients and 10 eyes of 10 unilateral patients) in RWD not consistent with the recommended surgical types in the CE group. The mean age was 23.41 ± 17.82 months and the mean axial length was 22.05 ± 1.20 mm in the 27 unmatched eyes. The mean visual acuity was 0.13 at 3 months after surgery and no complications occurred, which was not significantly different from the matched eyes in the CE group.

**FIGURE 2:**
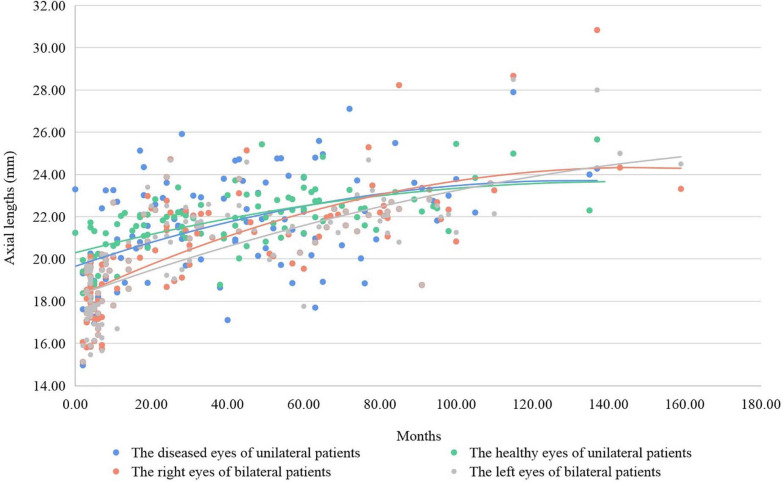
The axial lengths and growing fit lines of patients according to laterality. The axial lengths of unilateral patients were longer than those of bilateral patients before 7 years of age, and the axial lengths of bilateral patients became longer than those of unilateral patients after 7 years of age. The axial lengths of the healthy eyes of unilateral patients were longer than those of the diseased eyes before 6 years of age, and the axial lengths of the diseased eyes of unilateral patients became close to the healthy eyes of unilateral patients after 6 years of age.

In our research, the mean axial lengths were 19.04 ± 1.97 mm and 22.39 ± 2.04 mm in CE group and CE + IOL group, respectively, at baseline. The axial lengths of unilateral patients were longer than those of bilateral patients before 7 years of age. The healthy eyes of unilateral patients had longer axial lengths than the diseased eyes before 6 years old, and the axial lengths of the diseased eyes of unilateral patients became close to the healthy eyes after the age of 6 years (Figure 2). In the bilateral patients, the axial lengths became longer than those in the unilateral patients after 7 years of age.

## Discussion

Our study adopted RWD to validate the performance of real-world applications, which is a more efficient and objective method with lower risks. The consistency of the real surgical type and recommended surgical type was 94.16% in our study, which proved that the CC surgical decision model was reliable but still needed some improvement to obtain higher accuracy in real-world applications.

Childhood cataract is a rare disease with a mean morbidity of 4.24/10000 (Wu et al., 2016), and is a leading cause of childhood blindness (Lenhart et al., 2015). Surgery is the only effective treatment for most CC patients (Lim et al., 2017). However, the ocular structure of CC patients is abnormal and smaller than that of adults (Gopinath et al., 2013; Lin D. et al., 2016; Lin H. et al., 2016); consequently, CC surgery is difficult and challenging. On account of the rarity and exceptionality of CC patients, there is no consensus regarding the surgery time and surgery type for CC, which remain controversial worldwide. CCPMOH is a hospital-based national program with the largest clinical database of CC patients (Lin et al., 2015), based on data of 2421 patients from which, a ZOC team constructed a CC surgical decision model (Lin D. et al., 2019). The model can potentially serve as an objective basis for ophthalmologists to decide on surgical plans after more validations. Our study can further validate the real-world performance of the model, and ensure that it can be safely applied to clinical work.

In our study, we adopted the RWD of 398 eyes of 251 patients with CC. The consistency of the real surgical type and model-recommended surgical type was 94.16%, and the recommended surgical types for 27 eyes of 20 patients were not consistent with the real surgical types in the CE group. The 27 eyes had longer axial lengths at younger ages, and the model might recommend CE + IOL based on the data. However, in clinical work, considering that younger patients will have myopia shift and eye growth (Weakley et al., 2017; Liu et al., 2019) afterward and the high morbidity of postoperative complications (Kumar and Lambert, 2016), ophthalmologists usually choose CE surgery for young patients with long axial lengths. Aphakic patients can wear frame glasses and contact lenses to achieve necessary refractive correction before IOL implantation surgery (Russell et al., 2017). In addition, other ocular parameters, including anterior chamber depth and capsular size, can be included in the model to improve its accuracy and general applicability. As most IOLs are designed for adults and too large for children, severe deformities of the anterior and posterior capsula were observed in 30% of CC patients with IOL, which potentially led to IOL eccentricity and dislocation (Lin D. et al., 2019). The model with capsular-associated parameters may have a higher applied effectiveness.

In the unilateral patients, the axial lengths of diseased eyes were shorter than those of healthy eyes before 6 years of age and then the axial lengths of both eyes became closer. CC may lead to the developing suppression of the diseased eyes of unilateral patients at a younger age (Lin H. et al., 2016). After that, the diseased eyes may undergo rapid secondary development and approach the healthy eyes. The eyes of bilateral patients had a similar developing pattern to the diseased eyes of unilateral patients. The myopia shift of bilateral patients has a larger span (Liu et al., 2019), and most bilateral patients have longer axial lengths than both eyes of unilateral patients after myopia shift.

Rare diseases have a low morbidity of 0.5–1‰ (Europe, 2005; Haendel et al., 2020). It is usually difficult to conduct clinical control studies on rare diseases (Dong and Wang, 2016). The applications of RWD may solve the validation problems regarding medical models for rare diseases. The retrospective study we conducted achieved close-to-real and reliable test results based on RWD, which proved the feasibility and availability of our method. By collecting the RWD retrospectively from clinical centers, model validations can be efficiently accomplished. Compared to RWCS, our method has fewer executive risks for the indeterminacy of the real-world application of diagnosis and therapy assistance, which is safer and more generally accessible.

## Limitations

Some limitations of our research should be considered. RWD from more clinical centers is necessary to prove the general applicability of the surgical decision model. In addition, longer follow-up would contribute to the assessment of model efficacy. Additionally, the model may include more parameters to make it more precise.

## Conclusion

Our study has brought up a new method to validate the performance of medical assistant models. This is the first research to apply RWD to retrospectively evaluate a medical model and the results indicate the availability and feasibility of our new method, which may serve guidance for intelligent model evaluation for rare diseases.

## Data Availability Statement

The data is available from the corresponding authors upon reasonable request.

## Ethics Statement

The studies involving human participants were reviewed and approved by the Institutional Review Board of the ZOC at Sun Yat-sen University. Written informed consent to participate in this study was provided by the participants’ legal guardian/next of kin.

## Author Contributions

JC, DL, WC, and HL conceived and designed the experiments. JC, YX, LL, ZL, and WH collected the data. YX and AX cleaned the data. YX and DL performed the experiments and analyzed the data. YX wrote the manuscript. JC, FX, DL, and HL revised the manuscript. All authors read and approved the final manuscript.

## Conflict of Interest

The authors declare that the research was conducted in the absence of any commercial or financial relationships that could be construed as a potential conflict of interest.

## Publisher’s Note

All claims expressed in this article are solely those of the authors and do not necessarily represent those of their affiliated organizations, or those of the publisher, the editors and the reviewers. Any product that may be evaluated in this article, or claim that may be made by its manufacturer, is not guaranteed or endorsed by the publisher.
